# Temperance Restaurants

**Published:** 1899-10

**Authors:** 


### Temperance Restaurants.

One of the practical results of the International Anti-Alcohol Congress is the opening of a subscription for temperance restaurants by Dr. Legrain, physician-in-chief at Ville-Evrard. One subscriber gave $5,000, and a home-like restaurant has been opened in a poor neighborhood in Paris, with reading and lounging rooms. The prices are very low, and a single glass of wine or beer is served with the meals, none at other times, but tea, chocolate and coffee can be had at any time for from one to two and one-half cents. The success of the "Petits Repas Hygieniques," as it is called, has amazed even the most enthusiastic promoters.

Plaster-of-paris sets much more efficiently with sulphate of potash than with chloride of sodium. The potash salt may be used in any strength; the stronger the solution the quicker the setting.—*Atlanta Med. and Surg. Journal.*

